# On-line reductive cyclic voltammetric analysis of the anticancer agents mitomycin C, some of its degradation products and profiromycin

**DOI:** 10.1155/S1463924687000312

**Published:** 1987

**Authors:** H. H. J. L. Ploegmakers, M. J. M. Mertens, W. J. van Oort

**Affiliations:** Department of Analytical Pharmacy Faculty of Pharmacy State University of Utrecht Catharijnesingel 60 Utrecht 3511 GH The Netherlands


Journal of Automatic Chemistry/*Journal of Clinical Laboratory Automation*, Vol. 9, No. 4 (October–December 1987), pp. 149–157

### On-line reductive cyclic voltammetric analysis of the anticancer agents mitomycin C, some of its degradation products and profiromycin

**H. H. J. L. Ploegmakers, M. J. M. Mertens and W. J. van Oort**

*Department of Analytical Pharmacy, Faculty of Pharmacy, State University of Utrecht, Catharijnesingel 60, 3511 GH Utrecht, The Netherlands*

*An automatic, reliable, scanning reductive electrochemical detection system is described for on-line qualitative and semi-quantitative determination and characterization of electro-active compounds in effluents from high-performance liquid chromatographic (HPLC) systems. Applying a static mercury drop electrode, detection can be performed at constant potential for quantitative analysis (current range: 50 nA F.S.) at a routine base. Applying potential scans, qualitative analysis can be performed (current range: 0·5 μA F.S.). The system consists of easily obtainable commercial hardware, combined with commonly available chromatographic and electrochemical apparatus. Special attention has been paid to automatic, multi-reference-point component detection, data storage in a Ramdisk, machine language noise filtering, automatic half-wave potential and peak potential calculation and a high-resolution paper copy of the chromatograms and voltammograms. The system has been evaluated through analysis of mitomycin C, porfiromycin and the degradation products of mitomycin C from alkaline and acid hydrolysis.*

### Introduction

A computerized on-line cyclic voltammetric detection system after a high performance liquid chromatography (HPLC) has recently been described. Its characteristics were shown by qualitative (oxidative) analysis of a mixture of the antineoplastic agents etoposide (VP 16–213) and teniposide (VM 26) after chromatographic separation [1].

The software comprised an automatic detection system, which proved to be insensitive to noise smaller than a preset threshold value. In this way the effluent could continuously be scanned without storing any unwanted data. The analysis of the mixture of drugs by the described system is based on determination of the electrochemical parameters of the drugs. These parameters, based on the two-step oxidation of the phenolic hydroxyl group, proved to be in agreement with non-continuous analysis, as described by Holthuis *et al.* [2]. A striking difference between results of both studies proved to be the form of the voltammograms: waves in the case of continuous analysis, and peaks in normal non-continuous analysis.

Using the glassy carbon electrode (GCE) with cyclic voltammetry, the smallest amount of drugs to be analysed was 15 μg. This relatively high detection limit was caused by surface phenomena, producing large background currents.

The application of a static mercury drop electrode (SMDE) enables analysis of compounds in the negative potential range. It also provides advantages as regular refreshing of the electrode material. This avoids any pollution of the electrode-surface and ensures reproducibility of the electrochemical response. In fast-scanning methods the resulting background currents are lower than they would be with a glassy carbon electrode.

Several electro-active drugs (for example many of the common anticancer agents), are of particular interest for electrochemical investigations, because *in vivo* they need (enzymatic) oxidative or reductive activation. Examples of such processes are the oxidation of etoposide to a (stable) free radical, reported by Sinha [3 and 4] and the one-electron reduction of adriamycin to a semi-quinone radical, followed by glycolysis, enabling intercalation in DNA, described by Anne and Moiroux [5].

As a model compound for the development of an automatic system for reductive, qualitative analysis of drugs, using an HPLC/scanning electrochemical detection (ECD) system, the anticancer drug, mitomycin C, was selected. The electrochemical behaviour of mitomycin C has already been investigated in non-continuous analysis. It tends to produce a considerable number of electro-active degradation products and metabolites *in vivo*, which substantially hampers isolation of all products.

Structure analysis and solvolysis of mitomycin C has been reported by Stevens *et al.* [6] and Taylor and Remers [7], using UV, NMR and IR spectrometric analysis and degradation (pKa) studies.

Recently, Beijnen *et al.* [8] described the analysis of mitomycin C, using reversed-phase chromatography with UV detection, IR and mass spectrometry and non-continuous d.c. polarography.

Den Hartigh [9] reported the polarographic behaviour of mitomycin C, the bio-analytical applications of reversed-phase chromatography using electrochemical detection at constant potential and pharmacokinetics in cancer patients.

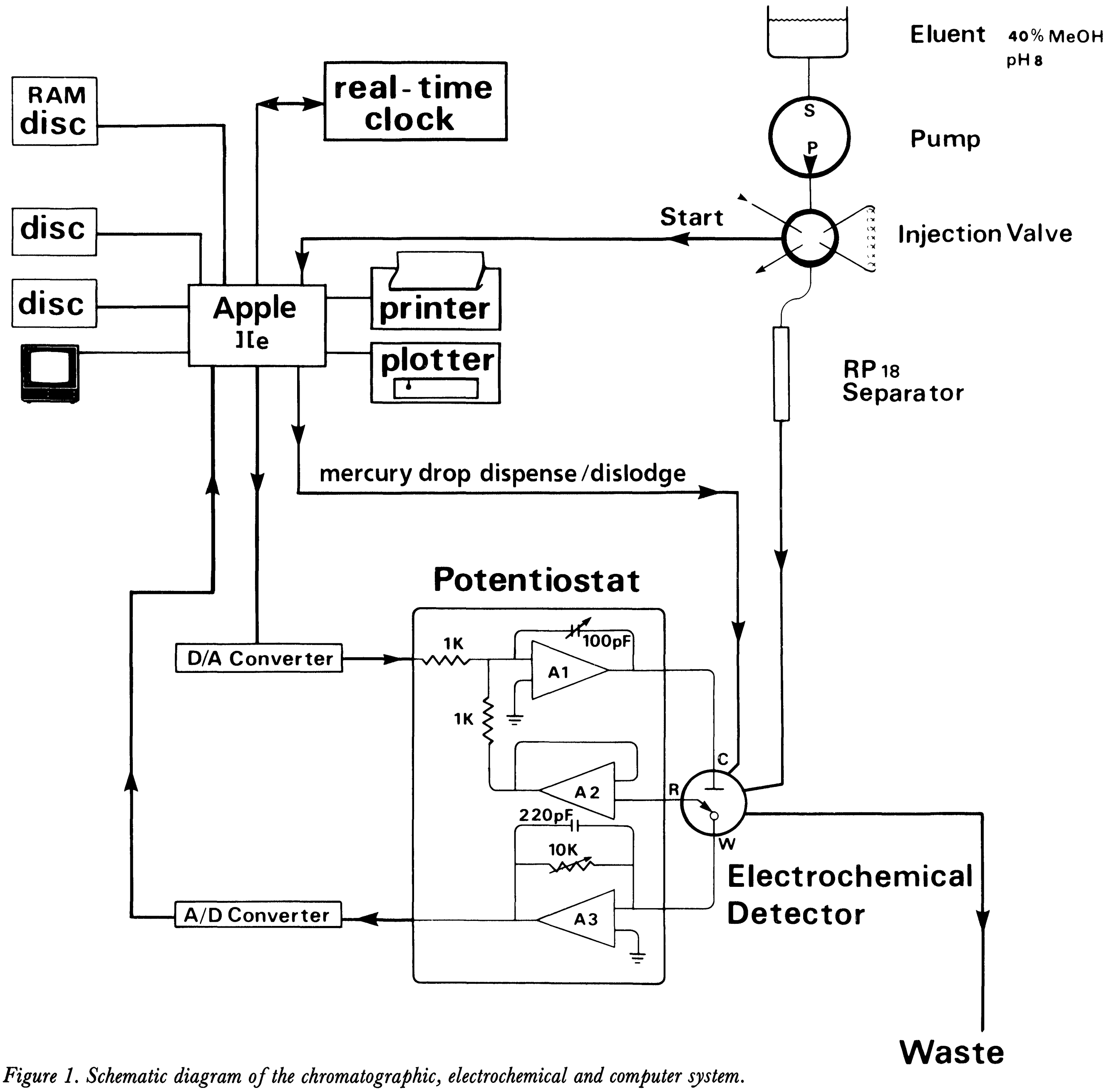

*Figure 1. Schematic diagram of the chromatographic, electrochemical and computer system.*

In analysing unstable compounds, results of non-continuous polarographic analysis, providing qualitative and quantitative information, are particularly difficult to interpret. Moreover, HPLC analysis with ECD at constant potential provides only quantitative information.

Especially, in *in vitro* modelling studies of *in vivo* processes, there is a strong need for qualitative information to be produced quickly. In this investigation, the combination of HPLC, ECD and potential scanning methods has been explored for this ability.

Computerized scanning methods after HPLC might be well-suited to separation, as well as production, of qualitative and semi-quantitative information, when only small amounts of drugs and (metabolic) degradation products are available.

### Experimental

### *Hardware requirements*

The heart of the on-line pilot-voltage-generating and data-acquisition system used is an Apple IIe microcomputer (figure 1).

The microcomputer is equipped with 64 Kbytes of on-board RAM and two 125 K Apple floppy disk drives. The micro was combined with an Axlon 320 K Ramdisk, a 12-bit 16-channel Digilog A/D converter, a 12-bit D/A converter (made by coupling a California Computer Systems 7720 parallel interface and an Analog Devices AD DAC80 D/A converter), a California Computer Systems model 7424 real-time clock, an Itoh 8510 parallel printer with screen-dump facility (Kronemuis APL 13 graphic printer interface), and a Hewlett-Packard 7470A serial plotter.

A Metrohm 641 VA-Detector (output: 1 V F.S.) and a Princeton Applied Research 310 polarographic detector were connected to this system. The chromatographic system consists of a 510 solvent delivery system, an U6K septumless injector (both from Waters Associates) and a LiChrosorb RP-18 analytical column (30 cm × 3·9 mm i.d. 10 μm).

### Chemicals

Mitomycin C was a gift from the Bristol-Myers Co. (Syracuse, New York, USA).

Porfiromycin was kindly supplied by Dr D. B. Borders, Lederle Laboratories (Pearl River, New York, USA)

The mobile phase consists of methanol/0·01 M phosphate buffer (pH 8) (40:60 w/w).

For the analysis of mitomycin C and porfiromycin, solutions of 0·87 mg/ml each in methanol were used.

The injected amounts were variable, depending on the application.

### Software and interfacing

Detector-computer interfacing is achieved by connecting annunciator outputs of the Apple to the rear-side connector, J1, of the Princeton Applied Research 310 detector: in this way it is possible to control the mercury drop knocker and the nitrogen stream.

The injector contains a switch, connected to an input on the game-connector of the Apple by a 74LS00 flip-flop debouncer in order to control and to detect the moment of injection.

The D/A converter is connected to the pilot voltage input of the potentiostat by an amplifier (gain: 10), enabling to set the desired potential of the working electrode.

The recorder output of the potentiostat is connected to the A/D converter by an amplifier/attenuator/offset circuit to adapt the input signal to the voltage range of the A/D converter.

After deaerating the solution in the detector cell for 10 min by nitrogen, the HPLC program is executed.

'HPLC' (table 1) is a program to perform HPLC with reductive electrochemical detection at constant potential at the SMDE. It investigates the number of components present in the sample and establishes the retention times of those substances.

Switching on the detector cell, the current is usually out of range (overload), due to stabilizing processes at the electrode surface. Therefore, the CLOCK program, a subroutine of the HPLC/ML program, enables resetting and restarting the real-time clock, the data storage and the high-resolution graphic screen after a stabilizing period without loosing the variables.

*Table 1. Description of the functions of the programs.*

| Language | Program name | Function |
|---|---|---|
| Basic | HPLC | H.p.l.c./e.c.d. at constant potential |
| Assembly | CLOCK | To reset and restart real time clock and data-storage in HPLC/ML |
| Assembly | HPLC/ML | Measuring, data storage, timing and drop dislodging |
| Basic | PARAMETER | Parameters for cyclic voltammetry |
| Assembly | DUMMY | To stabilize s.m.d.e. |
| Assembly | THRESHOLD | Detection of the passage of a compound through the detector |
| Assembly | DETERMINATION | To run a cyclic voltammogram of a compound in the detector |
| Basic | CALCULATION | Display of voltammograms on screen and production of a paper copy; to filter data and to calculate peak potential and 'half-wave potential' |

Applying the PAR 310 detector, each time a new drop is dispensed, the volume of the drop reaches its final volume very fast, and shortly after that, the capacity current reaches its minimum value (Bond and Jones [10]). Therefore, by inserting a software delay of 1 s after dispensing the mercury drop, the current can reach a constant value almost freed from capacity current. After this delay, 16 current measurements are executed and averaged, using a machine language routine. The result is stored into the memory and plotted on screen in the high resolution graphic mode.

The HPLC program also enables saving data as well as parameters on disk, smoothing the chromatogram, using a machine language 11-point parabolic filter routine, plotting the curves, calculating the retention times and plotting this information in combination with the peaks.

In a previous report, a cyclic voltammetric detection system in HPLC was described; this used a glassy carbon electrode [1]. The on-line cyclic voltammograms of etoposide and teniposide, obtained in this way, show waves. To detect a compound in the electrochemical cell in that system, it is sufficient to compare the current at a certain potential (for example +700 mV) with the corresponding current measurement of the blank scan: if the current value is higher then the corresponding blank value, increased with an user-defined threshold, it indicates a compound, passing the detector.

The on-line cyclic voltammograms of the degradation products of mitomycin C, decomposed at pH = 2, however, show peaks. In such a case, the current decreases at the end of the scan. Therefore, current comparison at a single potential value at the end of each scan, is not sufficient.

After stabilizing the system, using six dummy scans (DUMMY program), the THRESHOLD program is executed. After storage of a blank scan, every 100th current measurement is increased with a user-defined threshold value and stored in the memory. At scanning, each 100th current measurement is compared with the corresponding stored blank current value. If a current measurement is larger, it indicates that a compound is passing the detector. Then a detection flag is set. After completion of the scan, the computer checks the flag and if set, the program jumps to a measuring and storage DETERMINATION subroutine. This subroutine stores six scans, accompanied with real time into the Ramdisk. After completing this subroutine, the program returns to the THRESHOLD scanning and comparing mode.

As the voltammograms of the parent drugs and decomposition products show waves as well as peaks, the CALCULATION program is adapted with a peak potential calculation routine.

The continuous flow of the eluent against the fragile mercury drop sometimes blows off the drop from the capillary, resulting into a sudden current cut-off. Frequency of this process proved to be influenced by the flow rate, the composition of the mobile phase and the potential of the working electrode.

The computer can be used to compensate this event. In the CALCULATION program, all stored current values are compared with their preceding current measurement; if the difference is less than a user defined value, then a current cut-off is detected and this current value is discarded and replaced by its predecessor: in this way, the curve is smoothed partially.

Applying the CALCULATION half wave potential calculation subroutine, it is necessary to smooth the curve first. The parabolic filter method, written in Applesoft Basic, has previously been applied [1]. The software of this method can easily be programmed but it is time-consuming. Because of the favourable data handling, obtained in that way, a 6502 machine language 11-point parabolic filter program has been written. This program filters the data very quickly in the calculations.

All software is available from the authors on request.

### Results and discussion

### *HPLC with reductive ECD at constant potential*

Previously, several liquid chromatographic systems using polarographic detection have been described, for example by Rucki [11], Lloyd [12], Samuelsson *et al.* [13] and Scanlon *et al.* [14]. Van Oort *et al.* [15] evaluated a system consisting of a Princeton Applied Research 174 polarograph, equipped with an Princeton Applied Research 310 polarographic detector and an Y-t recorder. Figure 2(*a*) shows a chromatogram of a mixture of mitomycin C and porfiromycin, using ECD at constant potential, obtained with the computerized system, described in this paper. The retention times are listed in table 2.

*Table 2. Retention times, 'half wave potentials' and peak potentials of mitomycin C, porfiromycin and some degradation products of mitomycin C at pH 11·6 and pH 2.*

| Compound | Retention time | 'Half wave potentials' wave 1 | wave 2 |
|---|---|---|---|
| Mitomycin C | 4′ 10″ | −526 mV | |
| Porfiromycin | 4′ 40″ | −522 mV | |
| Oxygen | 5′ 36″ | not calculated | |
| 7-hydroxy-mitomycin C | 2′ 58″ | −568 mV | −846 mV |
| | | Peak potentials forward | reverse |
| Cis-2,7-diamino-1-hydroxymitosene | 4′ 54″ | −672 mV | −503 mV |
| Trans-2,7-diamino-1-hydroxymitosene | 3′ 54″ | −674 mV | −492 mV |
| Cis-2-amino-1,7-dihydroxymitosene | 3′ 10″ | −660 mV | −495 mV |
| Trans-2-amino-1,7-dihydroxymitosene | 2′ 54″ | −680 mV | −490 mV |

*Conditions of the HPLC experiments:*
*mobile phase: methanol/0·01 M phosphate buffer (pH = 8) (40:60 w/w); LiChrosorb RP-18 column; flow rate: 0·9 ml/min*
*Constant potential mode: potential: −700 mV vs. Ag/AgCl (3 M KCl) electrode*

| | | |
|---|---|---|
| *mitomycin C:* | *5 µg, sensitivity:* | *50 nA F.S.* |
| *porfiromycin:* | *5 µg,* | *50 nA F.S.* |
| *degradation at pH 11·6:* | *50 µl,* | *50 nA F.S.* |
| *degradation at pH 2:* | *50 µl,* | *50 nA F.S.* |

*Cyclic voltammetric mode: scan speed: 300 mV/s vs. Ag/AgCl (3 M KCl) electrode*

| | | |
|---|---|---|
| *mitomycin C:* | *10 µg, sensitivity:* | *0·5 µA F.S.* |
| *porfiromycin:* | *10 µg,* | *0·5 µA F.S.* |
| *degradation at pH 11·6:* | *50 µl,* | *0·5 µA F.S.* |
| *degradation at pH 2:* | *50 µl,* | *0·5 µA F.S.* |

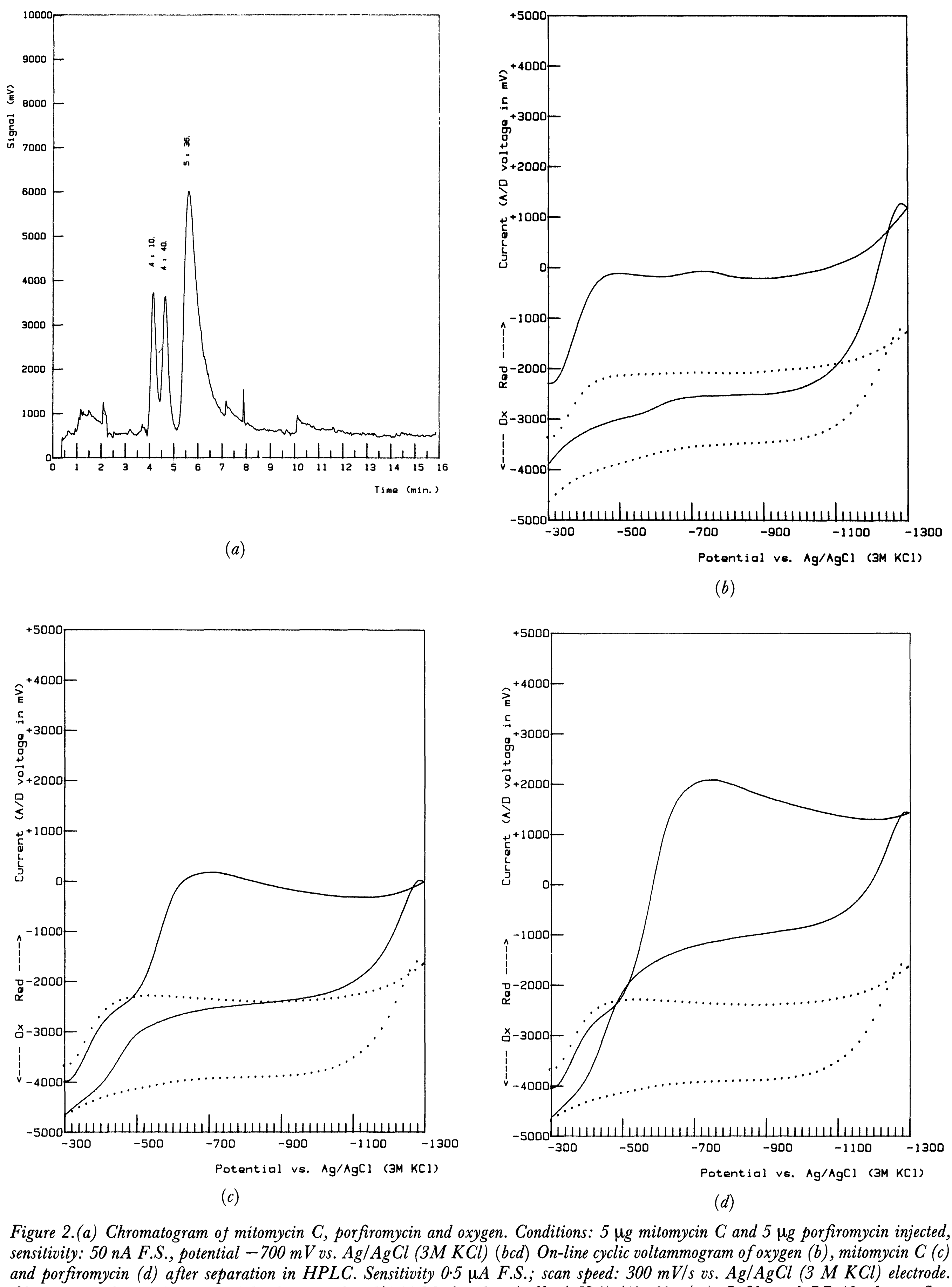

*Figure 2.(a) Chromatogram of mitomycin C, porfiromycin and oxygen. Conditions: 5 μg mitomycin C and 5 μg porfiromycin injected, sensitivity: 50 nA F.S., potential −700 mV vs. Ag/AgCl (3M KCl) (bcd) On-line cyclic voltammogram of oxygen (b), mitomycin C (c) and porfiromycin (d) after separation in HPLC. Sensitivity 0·5 μA F.S.; scan speed: 300 mV/s vs. Ag/AgCl (3 M KCl) electrode. Chromatographic conditions: mobile phase: methanol/0·01 M phosphate buffer (pH 8) (40 : 60 w/w); LiChrosorb RP-18 column; flow rate: 0·9 ml/min.*

Due to its reducibility, oxygen can interfere the detection, which depends on the applied detection potential. In the constant potential detection mode, the presence of oxygen in the mobile phase causes a high background signal, whereas the presence of oxygen in the sample causes a broad chromatographic peak (retention time: 5′ 36″), which may mask other peaks (figure 2[*a*]).

In the cyclic voltammetric mode, oxygen, present in the sample, appears as a wave, which may overload the potentiostat upon measuring at high sensitivities (figure 2[*b*]). The 'half-wave potential' of oxygen is about −380 mV in the applied mobile phase solution.

Hanekamp [16] describes an electrochemical scrubber, to remove the oxygen from the mobile phase. In this method relatively high buffer concentrations need to be applied to neutralize the produced hydroxyl ions.

Reim [17] reports the application of a post-column deoxygenator for liquid chromatography, based on the gas permeation of silicon rubbers.

In order to decrease the oxygen concentration, several authors add sodium sulphite to the blank solution in non-continuous polarographic experiments (Heyrovsky [18]) as well as the HPLC analysis with electrochemical detection at constant potential (den Hartigh [9], Persson and Rosen [19]). This method leads to satisfactory results, using both modes, if the pH of the solution is larger than pH 7.

As sulphite is reduced at potentials more negative than −700 mV (depending upon pH), this method is not acceptable in the cyclic voltammetric mode, whereas high pH-values may affect the stationary phase of the HPLC column.

Most of the oxygen can also be satisfactory removed in a more classical way; by purging nitrogen through the stock solution of the mobile phase and samples (Meites [20]). This method was chosen for the present system, yielding an almost flat blank voltammogram (see dotted line in figure 2[*b*]), in the applied sensitivity range.

*HPLC with cyclic voltammetric detection at the static mercury drop electrode*

In order to evaluate the HPLC/ECD system in the cyclic voltammetric mode, a mixture of mitomycin C and porfiromycin was injected. In non-continuous d.c. polarographic experiments of solutions at pH 8, using the SMDE, mitomycin C, as well as porfiromycin, show only one reduction wave. This electrochemical activity is caused by the reduction of the quinone group, according to a double electron-proton uptake mechanism (e H e H) [9].

In on-line cyclic voltammetry after HPLC, using the same electrodes, i/E curves of mitomycin C and porfiromycin also show a single reduction wave in the forward scan (see figure 2 [*c*] and [*d*] and table 2), provided with a small maximum.

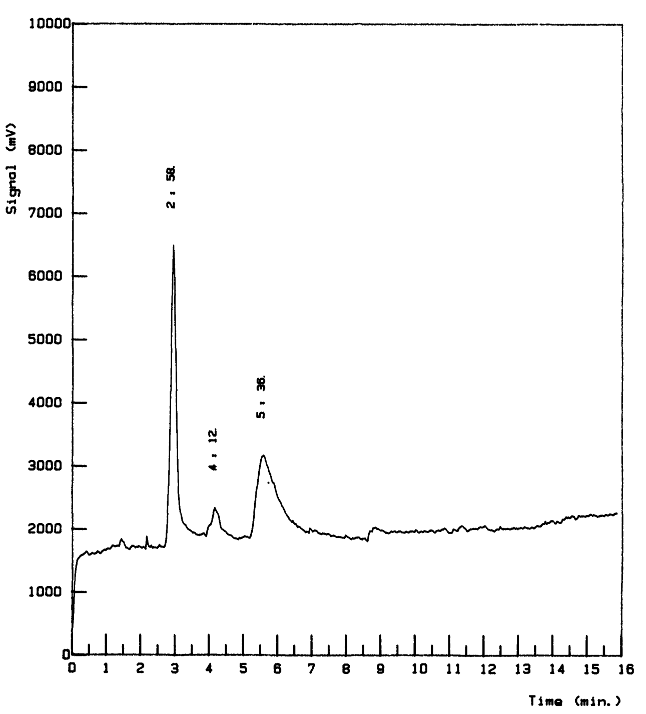

*Figure 3(a). Chromatogram of the decomposition product of mitomycin C (7-hydroxymitomycin) at pH 11·6. Conditions: 50 µl injected, sensitivity 50 nA F.S., potential:- 700 mV versus Ag/AgCl (3M KCl)*

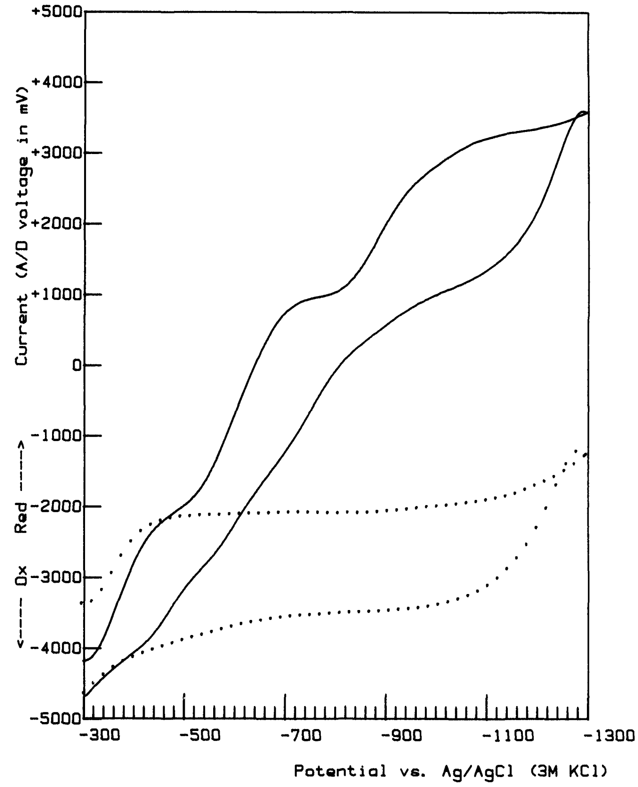

*Figure 3(b). On-line cyclic voltammogram of the decomposition product of mitomycin C at pH 11·6 after separation in h.p.l.c. Conditions: 50 µl injected; sensitivity 0·5 µA F.S.; scan speed: 300 mV/s vs. Ag/AgCl (3 M KCl) electrode. Chromatographic conditions as figure 2.*

As the limiting current in flow systems is expected to be governed by diffusion as well as convection (Bond [21]), the term 'half wave potential' has been used in table 2, to indicate the difference between this expression and the classical term $E_{1/2}$, used in conventional d.c. polarography at a dropping mercury electrode.

The decomposition of mitomycin C at pH = 11·6 has recently been described by Beijnen *et al.* [8]. Mitomycin C was degradated by mixing 100 μl of a stock solution (8·7 mg in 10·0 ml MeOH) with 1·00 ml 0·01 M sodium phosphate buffer (pH = 11·6). After 1 h incubation (at room temperature and in the dark), the reaction was terminated by changing the pH to pH = 8 using 0·1 N hydrochloric acid; 50 μl of the reaction mixture was injected into the HPLC system.

The results are shown in figure 3(*a*) and (*b*). Figure 3(*a*) shows mitomycin C to be almost totally converted into another, also electro-active, substance. Beijnen *et al.* [8] showed this substance to be 7-hydroxy-mitomycin C.

The on-line cyclic voltammogram of this new compound (figure 3[*b*]) indicates two reduction steps (see table 2), which is in accordance with non-continuous experiments [9].

Figure 3(*a*) and (*b*) show the advantage of the cyclic voltammetric detection system after HPLC, presented here, over the batch-wise method: the computerized on-line scanning system enables one compound to be distinguished from another, showing two reduction steps, or a mixture of two compounds each with one reduction step. In addition, parameters like reversibility, consecutive reactions and time effects can be quickly established.

Taylor *et al.* [7] and Stevens *et al.* [6] have described the degradation of mitomycin C in acid solutions. After evaporating the methanol of 50 μl mitomycin C solution 8·7 mg/10 ml MeOH) at room temperature, the reaction is started by dissolving the mitomycin C in 1·00 ml perchloric acid solution (pH = 2). It is necessary to remove methanol, as it can act as a nucleophilic reagent, like water.

The products arising in first instance, are mainly cis-2,7-diamino-1-hydroxymitosene and a small amount of trans-2,7-diamino-1-hydroxymitosene and subsequently, small amounts of the cis-2-amino-1,7-dihydroxymitosene and trans-2-amino-1,7-dihydroxymitosene. [8]

The chromatogram of the reaction mixture with reductive ECD at constant potential is shown in figure 4; the retention times of these compounds are listed in table 2.

The chromatogram also shows that, at the applied sensitivity scale, oxygen (retention time: 5′ 38″) can be removed sufficiently from a sample by purging with nitrogen.

In the scanning voltammetric detection mode, using the SMDE, the voltammograms of the degradation products show peaks in the forward and the reverse scan (figure 5[*a*]–[*d*]).

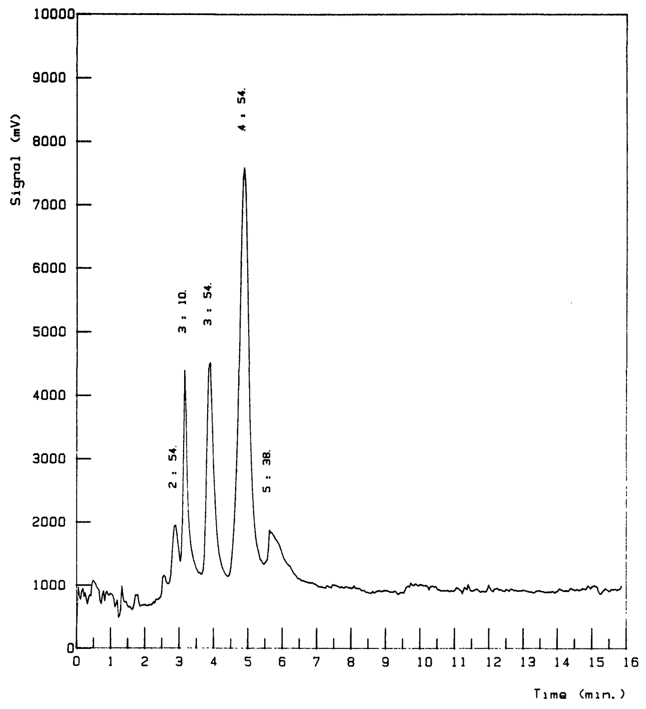

*Figure 4. Chromatogram of the decomposition products of mitomycin C at pH 2. Conditions: 50 μl injected, sensitivity 50 nA F.S., potential:- 700 mV vs. Ag/AgCl (3M KCl). Chromatographic conditions as figure 2.*

All compounds show the comparable electrochemical behaviour in forward and reverse scan whereas the peak potentials have almost identical values (table 2). Differences are noticed in ratios of the peaks heights in forward and reverse scans.

It is clear, that such small differences can not be noticed in non-continuous polarographic analysis of a mixture.

The system presented here, enables monitoring of the chemical degradation of 40 μg of mitomycin C into two main compounds and two additional products and characterizing the electrochemical properties of these products quickly and on-line.

### Conclusions

The cyclic voltammetric detection system, using the SMDE, described in this paper, has different and, in some respects, advantageous properties compared with the cyclic voltammetric detection system, using the glassy carbon electrode (GCE), described earlier [1].

In that cyclic voltammetric system with the GCE, the minimum amount needed for qualitative analysis, proved to be about 15 micrograms of etoposide. The reason for that unsatisfactory result proved to be the large background current, resulting from application of a GCE in fast potential sweep systems.

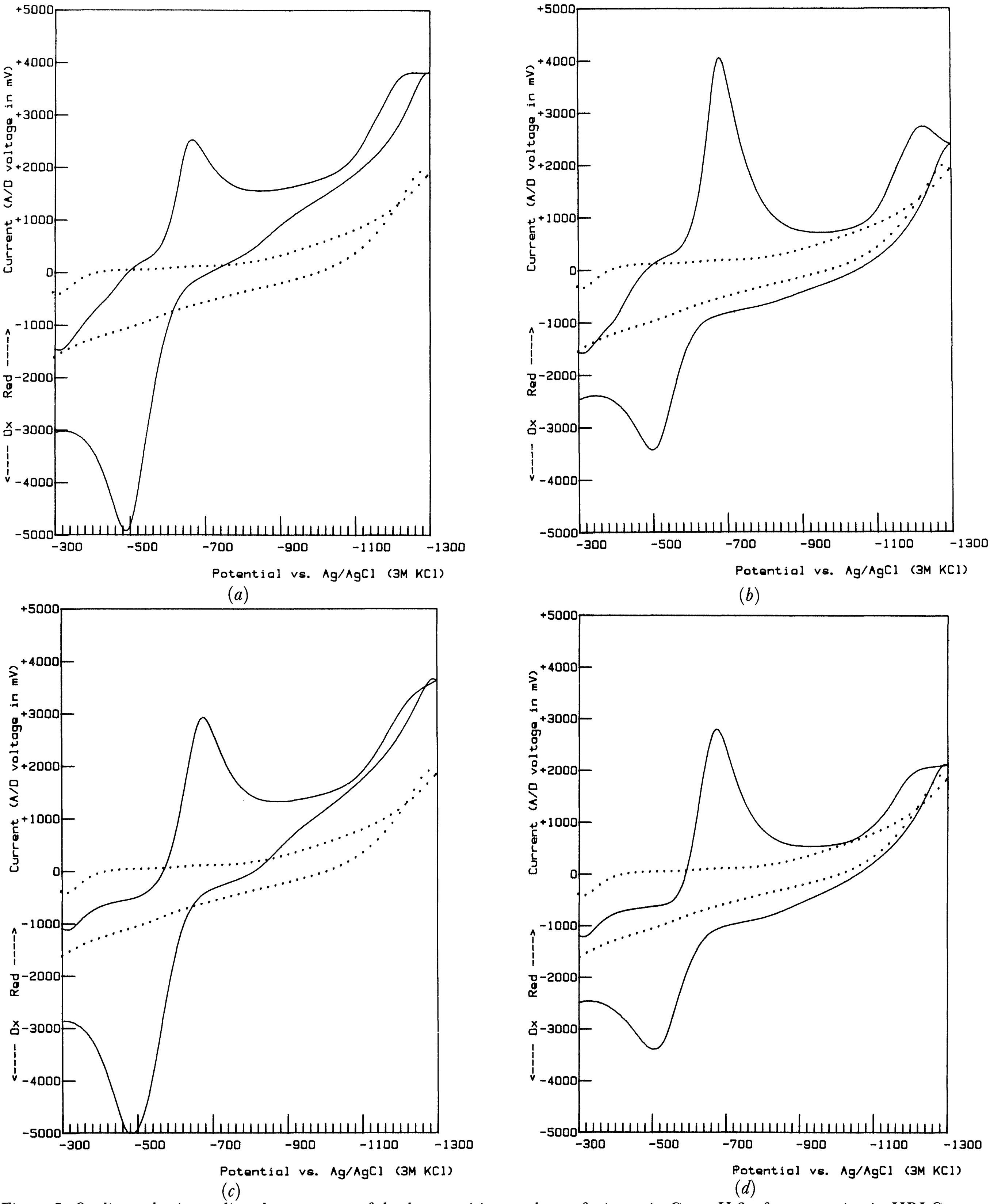

*Figure 5. On-line reductive cyclic voltammogram of the decomposition products of mitomycin C at pH 2 after separation in HPLC.*
*(a) Trans-2-amino-1,7-dihydroxymitosene.*
*(b) Cis-2-amino-1,7-dihydroxymitosene.*
*(c) Trans-2,7-diamino-1-hydroxymitosene.*
*(d) Cis-2,7-diamino-1-hydroxymitosene.*
*Conditions: 50 μl injected; sensitivity 0·5 μA F.S.;*
*scan speed: 300 mV/s vs. Ag/AgCl (3 M KCl) electrode.*
*Chromatographic conditions as figure 2.*

Using the static mercury drop electrode in the constant potential mode, the capacity current is decreased very quickly after dispensing each new drop, allowing measurement at 50 nA F.S. This feature also enabled application of SMDE in dynamic systems.

In the fast scanning mode, the minimum amount of mitomycin C or porfiromycin, to record a complete interpretable cyclic voltammogram proved to be 200 nanograms. Drugs and metabolites are regularly present at such levels in plasma or urine from patients.

Although the mercury working electrode is relatively fragile in flow systems, the developed software enables correcting for possibly preliminary dislodged mercury drops and decreasing the noise level.

Oxygen, dissolved in the mobile phase and samples, tends to interfere, but its influence can be diminished sufficiently by purging nitrogen through the mobile phase and samples and occasionally by misusing its known retention time.

In addition, the occurrence of real peaks in the cyclic voltammetric curves, obtained with the SMDE, are easier to interprete and to compare with batch-wise experiments than waves, observed in the oxidative system.

In future, the system will be used to investigate the electrochemical properties of other anticancer agents and possible metabolites in biological samples and reaction-mixtures and to investigate activation mechanisms of these drugs *in vivo*. For biomedical analysis of selected drugs, this technique can provide essential information, in addition to HPLC with scanning u.v. detection.

### Acknowledgement

The authors like to thank Mr J. Teeuwsen for his technical support and advice.

### References

1. Ploegmakers, H. H. J. L., Mertens, M. J. M. and van Oort, W. J., *Analytica Chimica Acta*, **174** (1985), 71.
2. Holthuis, J. J. M., van Oort, W. J., Romkens, F. M. G. M., Renema, J. and Zuman, P., *Journal of Electroanalytical Chemistry and Interfacial Electrochemistry*, **184** (1985), 317.
3. Sinha, B. K., Trush, M. A. and Kalyanaraman, B., *Biochemical Pharmacology*, **32** (1983), 3495.
4. Sinha, B. K., *Journal of Biological Chemistry*, **258** (1983), 796.
5. Anne, A. and Moiroux, J., *Nouveau Journal de Chimie*, **9** (1985), 83.
6. Stevens, C. L., Taylor, K. G., Munk, M. E., Marshall, W. S., Noll, K., Shah, G. D., Shah, L. G. and Uzu, K., *Journal of Medicinal Chemistry*, **8** (1965), 1.
7. Taylor, W. G. and Remers, W. A., *Journal of Medicinal Chemistry*, **18** (1975), 307.
8. Beijnen, J. H., den Hartigh, J. and Underberg, W. J. M., *Journal of Pharmaceutical and Biomedical Analysis*, **3** (1985), 71.
9. den Hartigh, J., Thesis, State University of Utrecht, 1986.
10. Bond, A. M. and Jones, R. D., *Analytica Chimica Acta*, **121** (1980), 1.
11. Rucki, R. J., *Talanta*, **27** (1980), 147.
12. Lloyd, J. B. F., *Analytica Chimica Acta*, **154** (1983), 121.
13. Samuelsson, R., O'Dea, J. and Osteryoung, J., *Analytical Chemistry*, **52** (1980), 2215.
14. Scanlon, J. J., Flaquer, P. A., Robinson, G. W., O'Brien, G. E. and Sturrock, P. E., *Analytica Chimica Acta*, **158** (1984), 169.
15. van Oort, W. J., den Hartigh, J. and Holthuis, J. J. M., *Proceedings of Symposium on Electrochemical Detection in Flow Analysis*, Ed. Pungor, E., Matrafured (Akademia Kiado, Publishing House of the Hungarian Academy of Science, Budapest, 1982), p. 365.
16. Hanekamp, H. B., Voogt, W. H., Bos, P. and Frei, R. W., *Analytica Chimica Acta*, **118** (1980), 81.
17. Reim, R. E., *Analytical Chemistry*, **55** (1983), 1188.
18. Heyrovsky, J., in *Polarographisches Praktikum* (Springer Verlag, Berlin, 1960), p. 69.
19. Persson, B. and Rosen, L., *Analytica Chimica Acta*, **123** (1981), 115.
20. Meites, L., in *Polarographic Techniques* (Interscience Publishers, New York, 1965), p. 89.
21. Bond, A. M., in *Modern Polarographic Methods in Analytical Chemistry* (Marcel Dekker, New York, 1980), p. 227.

**THE INTERNATIONAL CHEMICAL LABORATORY CONFERENCE: MANAGING INTO THE FUTURE**

*To be held in Zurich, Switzerland from 3 to 5 February 1988.*

Organized by the Management Centre Europe, the conference will include the following issues:

Economic and industrial outlook and prospects for the chemical industry
External controls – political, institutional and social
Global perspectives
Growth through established strengths and product innovation
Focusing R & D resources
New technologies: potential growth for the industry

The meeting has been arranged for managers in chemical and associated industries with the following objectives:

To identify and address key issues in managing a chemical business in today's environment.

To demonstrate the approach to maintaining growth and profitability by exploiting core strengths, methodically identifying new products by concentrating on customer needs and successfully managing R & D.

To examine some of the key issues impacting the future of the chemical industry including the management and information technology issues.

*Details from Management Centre Europe, rue Caroly 15, B 1040 Brussels, Belgium. Tel.: 32/2/5161911.*

